# Co-occurring microbial guilds in pig fecal microbiota: key drivers and effects on host performance

**DOI:** 10.1186/s12711-025-00979-x

**Published:** 2025-06-04

**Authors:** Ioanna-Theoni Vourlaki, Raquel Rio-Lopez, Adrià Clavell-Sansalvador, Lino C. Ramírez-Ayala, Maria Ballester, Juan P. Sanchez, Miriam Piles, Raquel Quintanilla, Angela C. da Fonseca de Oliveira, Leandro Batista Costa, Antoni Dalmau, Yuliaxis Ramayo-Caldas

**Affiliations:** 1 Animal Breeding and Genetics Program, IRTA, Torre Marimón, 08140 Caldes de Montbui, Barcelona Spain; 2 Animal Welfare Subprogram, IRTA, 17121 Monells, Girona, Spain; 3 Graduate Program in Animal Science, School of Medicine and Life Sciences-Pontifícia, Universidade Católica do Paraná (PUCPR), Curitiba, Brasil

## Abstract

**Background:**

The pig gut microbiota is a complex ecosystem composed of microbial guilds that remain largely unexplored. Here we decomposed the pig fecal microbiota of two cohorts of 648 healthy Duroc pigs during the transition (n = 400) and growing finish (n = 248) periods in co-occurring bacterial guilds defined as pig enterosignatures (ES).

**Results:**

Our results indicate that fecal microbial ecosystems can accurately be described by combinations of at least six ES, driven by the *Prevotella* (ES-Prev), *Treponema* (ES-Trep), *Lactobacillus* (ES-Lact), *Clostridium* (ES-Clost), *Streptococcus* (ES-Strep), and *UBA2810* (ES-UBA2) genera. We observed a dynamic shift with age in the composition of ES, where ES-Prev, ES-Strep, and ES-Lact seem to be core components. Our results suggest partial genetic control by the host, with heritabilities of ES composition ranging from 0.24 to 0.36. Furthermore, our findings indicate that stress on the host is associated with assembly of the ES, decreasing ES-Lact abundance, and increasing prevalence of ES-Strep. We noted a positive association of ES-Prev with growth rate at 60-days, which later evolved to become negative, impacting feed efficiency during the growing period. Remarkably, a negative association of the abundance of ES-Lact with levels of hair cortisol was also found during this period.

**Conclusions:**

Our findings provide novel insights into the pig gut microbiota and reveal novels associations with relevant porcine physiological and performance traits. Moreover, while the ES concept has proven valuable in dissecting microbial communities into assemblies of underlying microbial guilds, our results emphasize the relevance of customizing microbial interventions strategies based on the nutritional and health requirements at each stage of the porcine production cycle.

**Supplementary Information:**

The online version contains supplementary material available at 10.1186/s12711-025-00979-x.

## Background

The gut microbiota plays a significant role in mediating key processes associated with global pig production, one of the largest sectors of the livestock industry [1, 2]. Therefore, deeper knowledge of the genetic basis of gut microbiota and its relationship to genetic and environmental determinants is crucial for more sustainable and improved livestock production. The microbial communities of the gastrointestinal tract (GIT) form a diverse ecosystem, comprising microorganisms from various kingdoms, including bacteria, archaea, protozoa, fungi, and the virome [3]. Moreover, GIT microbial diversity and composition varies depending on several factors, such as diet, age, geography, season of the years, and host genetics [4–8].

In pigs, many studies have focused on taxonomic decomposition of the GIT microbiome, including the identification of distinct taxonomic levels that are the most prevalent across samples. Particularly, by analysing the relative abundances of genera, samples can be classified into categorical clusters known as enterotypes (ET) [9]. ET represent distinct gut microbiome configurations, characterized by dominant microbial taxa and specific metabolic pathways, which influence host physiology. The identification of porcine ET has been documented, providing insights into how gut microbiota composition can impact host performance. Analysis performed on a cohort of 518 Large White piglets reported two ET, driven by either the *Ruminococcus* and *Treponema* or the *Prevotella* and *Mitsuokella* genera [10]. Another study [11], based on a crossbred pig population (Duroc males with Large White × Landrace or Landrace × Large White sows), found two distinct ET for weaning animals, a *Prevotella*-enriched and an *Escherichia*-enriched communities. Likewise, two different sets of clusters were identified in a longitudinal analysis of microbiota from post-weaning to finishing [12]. At 52 days, reported ET were driven by *Lactobacillus* and *Prevotella*-*Sarcina,* while from 99 to 154 days the driver genus were *Lactobacillus* and *Turicibacter*-*Clostridium*. While clustering methods used to identify ET have contributed to understanding of the pig microbiota, their implementation assumes that each of the identified communities is driven by single distinct dominant phylogenetic groups. This approach fails to capture the co-existence of bacterial guilds under various conditions that can affect the assembly of the microbial ecosystem. Furthermore, the allocation of samples to each cluster results in a categorical variable, which oversimplify the inherent complexity of microbial ecosystems. Despite these limitations, previous studies have reported association of ET with porcine performance traits, including growth, body weight, and average daily gain [10–13].

Recently, Frioux et al. [14] introduced the complementary concept of enterosignatures (ES), defined as a microbial signature of commonly co-occurring bacterial guilds. In gut microbiota research, a “guild” refers to a group of microorganisms that exhibit consistent co-abundance patterns and that likely collaborate in performing a shared ecological function [15]. Compared to ET, which classify samples into discrete microbial ecosystem types, the proposal by Frioux et al. [14] is able to identify assemblies of underlying microbial guilds that explained most of the gut microbial variance. While the ET concept assigns each sample to a single, static cluster, it overlooks the complex, co-occurrence patterns that shape microbial community structures. In contrast, the ES approach considers the intrinsic ecosystem information by providing a proportional representation of bacterial assemblages, rather than a simple categorical classification. As a result, the microbial composition in each sample is represented by an assembly of different bacterial guilds, with their relative abundance explicitly quantified. Furthermore, for each bacterial guild, the ES approach reveals the composition and contribution of the individual genera that compose it, offering a dynamic and detailed understanding of the microbial community. Frioux et al. [14] found that the human gut microbiome can be characterized as a combination of at least five complementary ESs. Decomposition of human gut microbiome was performed by non-negative matrix factorization (NMF) [16], a multivariate approach where the latent variables are continuous rather than categorical. While this approach has been applied to human gut microbiome, to the best of our knowledge, the occurrence of ESs in the pig microbiota has not yet been documented.

Here, we explore the existence of ESs in pig faecal microbiota. The primary objective of our study was to decompose faecal pig microbiota into bacterial guilds. Our focus extends to investigate the composition of ESs, identifying key drivers and determining whether the ESs exhibit stable or dynamic fluctuations across time. Additionally, we aim to explore the diversity of ES between host and environmental determinants to reveal their association with host performance.

## Methods

### Experimental design

Animal care and experimental procedures were carried out following national and institutional guidelines and were approved by the IRTA Ethical Committee. A total of 648 healthy Duroc pigs from the same commercial genetic line, at two different ages, and from two farms were employed. A total of 400 weaned piglets (201 males and 199 females) distributed across six batches were sampled in a commercial farm at 60 ± 8 days of age after 4 weeks. All pigs received the same transition-based diet. Refer to Ramayo-Caldas et al. [2] and Ballester et al. [17] for more information about animal management and data collection. The remaining 248 pigs (119 castrated males and 129 females) were raised under intensive standard conditions at the IRTA experimental farm (IRTA, Monells, Spain) (see Additional file 1 Figure S1).

### Sample collection

Faecal samples of the 400 piglets were collected at 60 ± 8 days of age, and faecal and hair samples of the remaining 248 pigs were collected at 190 ± 10 days of age, when pigs were fed a standard finishing diet. For 134 of the 248 pigs, BW (kg) and average daily feed intake (kg) were recorded using both a weighing scale and electronic feeding stations during the experimental period. These comprehensive datasets were employed to calculate Average Daily Gain (ADG), Feed Conversion Ratio (FCR), and Residual Feed Intake (RFI). Moreover, 96 of the 248 growing pigs, comprising 48 castrated males and 48 females, were allocated into eight pens of 12 animals. Five of the 96 pigs were excluded prior to distribution into experimental groups due to prior antibiotic use, while the remaining 91 were distributed across two treatments.: T1—control group (N = 44), and T2—stress group (N = 47), in which social stress was induced by mixing the pigs three times during the growing-finishing period (see Additional file 1 Figure S1). Pigs in the control group were not mixed.

Cortisol (CORT) levels in hair samples of these pigs were analysed at the end of the experiment (190 days-old) as follow: 150 mg of hair was weighted from each hair sample and placed into a 50-ml conical tube. After three washes with 3 ml of isopropanol, all samples were left to dry in an extractor hood during 12 h. Dried hair samples were cut into 2–3 mm pieces using scissors, and 50 mg were transferred into 2 ml eppendorf. One ml of methanol was added to each sample and incubated 18 h at 37 °C under moderate shaking (100 rpm). After incubation, extracted samples were centrifuged at 7000*g* for 2 min and 700 µl of supernatant was transferred to a new 1.5 ml tube. The supernatant was placed into a speed vac for 2 h to evaporate the methanol. The dried extracts were stored at − 20 °C until analysis. Total concentrations of CORT were measured by ELISA kit (Cusabio Technology LLC., Bionova, Spain), using dried samples reconstituted with 210 µl of sample diluent. Samples were quantified by reference to standard curves constructed with known concentrations of pig cortisol dilutions of the Standard. Absorbance was read at 450 nm using an ELISA plate reader (Bio-Rad) and analysed using the Microplate manager 5.2.1 software (Bio-Rad).

### Microbial DNA extraction, sequencing, and bioinformatics analysis

DNA was extracted from the faecal samples with the DNeasy PowerSoil Kit (QIAGEN) and sent to the Keck Center at the University of Illinois for Fluidigm sample preparation and paired-end (2 × 250 nt) sequencing on an Illumina NovaSeq (Illumina, San Diego, CA, USA). The 16S rRNA gene fragment was amplified using the primers V3_F357_N: 5ʹ-CCTACGGGNGGCWGCAG-3ʹ and V4_R805: 5ʹ-GACTACHVGGGTATCTAATCC-3ʹ. Sequences were analysed with Qiime2 [18] and barcode sequences, primers, and low-quality reads (Phred scores of < 30) were removed. The quality control also trimmed sequences based on the expected amplicon length and removed chimaeras. Afterwards, sequences were processed into Amplicon Sequences Variants (ASVs) at 99% identity. ASVs present in less than two samples and representing less than 0.0001% of the total counts were filtered out. Samples with less than 10,000 reads were also excluded. ASVs were classified to the lowest possible taxonomic level based on a primer-specific trained version of GreenGenes2 database [19].

### Identification of enterosignatures

Following Frioux et al. [14], to evaluate the existence of ES, NMF was applied to the normalized genera relative abundance table. The latter was created by retaining genera that were present in more than 20% of the samples and normalizing the abundance data in each column, representing an individual sample, by dividing by its sum, such that all columns had a total sum of 1 and positive values.

The NMF decomposed the abundance table in two new ones: **W**, defined as the weight of the genera in ESs, and **H**, defined as the presence of ESs across samples (see Additional file 2 Figure S2). Particularly, **W** represents the contribution of the genera (n in rows) to the resulted ESs (k in columns). The optimal number of ESs was evaluated by performing a nine-fold bi-cross validation [20], as suggested for choosing the best rank in outer product models such as NMF. We implemented bi-cross validation using the python script “bicv_rank.py” from the repository https://gitlab.inria.fr/cfrioux/enterosignature-paper of Frioux et al. [14]. For each of the 9-folds, the model used 8 of the folds as the training set, while the performance was validated on the remaining data set. This process was repeated 200 times for the number of ESs, k, ranging from 2 to 30, randomly shuffling the matrix for each repetition. The optimal k of ES was determined by tracking the derived evaluation metrics corresponding to each ES, such as reconstruction error, explained variance, and cosine similarity. NMF was applied using the python scripts of Frioux et al. [14] and available in repository https://gitlab.inria.fr/cfrioux/enterosignature-paper. Their implementation was executed via the terminal using Python 3.9 [21], and packages SciPy v1.7.3 [22] and Scikit-Learn v0.24.1 [23]. The non-negative matrix factorization (NMF) was run for 100 iterations, with a maximum of 2000 iterations per run. The model used random initialization, a regularization ratio of 1, the multiplicative update solver, and the Kullback–Leibler divergence as the beta-loss function. Regularization was applied to both the **W** and **H** matrices.

### Enterosignature analyses

After reducing the putative number of clusters, a second step of the ESs evaluation was done using the *enterosignatures_nmf.py* script (https://gitlab.inria.fr/cfrioux/enterosignature-paper) for each of the two data sets (400 and 248 samples). This script used the previously estimated optimal ESs k as a hyperparameter. In addition to this evaluation, we ran the pipeline for a range of k from 2 to 10, keeping the output evaluation scores. The driven genus in each k-ESs was identified as the genus that had the highest contribution value across all genera, while the assignment probability of genera to ESs was retrieved by row-wise normalization of the **W** matrix. Furthermore, the normalized **H** matrix was used to accurately describe the relative contribution of each ES, which—when multiplied by 100—represents the percentage of explained variance within a sample. In addition, this matrix served to explore associations between ES composition and host phenotypic covariates.

### Determinants of ES and association with host performance

We aimed to explore the determinants influencing ES composition, considering both host-specific and environmental factors, and to examine associations between ES and host traits. Our dataset comprised 648 samples gathered at two ages (N = 400 at 60 days vs N = 248 at 190 days of life), from two farms (N = 400 commercial vs N = 248 experimental facilities), and from diverse experimental setups. Consequently, metadata information varied between the samples. To address this, we conducted separate analyses for the 400 and 248 samples based on the available information as outlined below:

The composition of ESs for the 400 piglets was first explored in relation to batch and sex. For the batch, samples were distributed in six different groups. For sex, the data contained 201 males and 199 females. For these factors, pie plots were generated to demonstrate the minimum number of ESs needed to explain most of the variance within each factor level, as well as the ES distribution across levels. Therefore, ESs were ranked in descending order based on their explained variance, and the cumulative explained variance was progressively calculated to identify the minimum number of ESs accounting for at least 90% of the total variance. The frequency of specific ES that appeared in the minimum number of ESs was also estimated. Associations of ES abundance with BW were investigated across the 400 samples using package ggplot [24] in R [25], with the geom_smooth (method “lm”) function to add a regression line. Note that prior to testing the ES association to BW, the latter was corrected by using residuals from fitting age as covariate using the *lm* model. The analyses were performed using a univariate approach, i.e. one ES at a time for each phenotype.

For the 248 190-day-old pigs raised under experimental conditions, our initial focus was on examining the diversity of ESs separately for the 119 castrated males and 129 females. We then conducted two following independent analyses to explore the association of ESs with quantitative performance and physiological traits:

1. The dataset of 134 pigs was utilized to investigate the relationship of ESs with ADG and feed efficiency traits (FCR, RFI).
2. The impact of stress challenge on ES distribution was evaluated based on the dataset of 47 stress vs. 44 control pigs. This dataset was also employed to assess the association of ES abundance with levels of hair cortisol.

### Genotyping, and estimation of genetic parameters

After evaluating host-specific and environmental factors affecting ES, the potential genetic impact of the host was investigated using the 400 samples collected at 60 ± 8 days of age. The use of this larger dataset than the growing pig data set was expected to improve the power and the accuracy of estimates of heritability. The Porcine 70 k GGP Porcine HD Array (Illumina, San Diego, CA) was used to genotype the 400 animals. The quality control excluded SNPs with minor allele frequencies < 5%, rates of missing genotypes above 10%, and SNPs that did not map to the porcine reference genome (Sscrofa11.1 assembly). The final SNPs data set consisted of 41,131 markers and 400 individuals. The narrow-sense heritability of ESs was estimated by applying the Reproducing Kernel Hilbert Space (RKHS) approach [26] from the BGLR package [27], separately for each ES using the following model:

1 $$ {\mathbf{y}}_{{{\mathbf{ES}}}} = {\mathbf{X}}{{\varvec{\upbeta}}} + {\mathbf{u}} + {\mathbf{e}} $$

where ${\mathbf{y}}_{\mathbf{E}\mathbf{S}}$ is the vector of individual ES abundances, ${\varvec{\upbeta}}$ is the vector of the systematic (fixed) effects of batch, sex, and the covariate of age, and $\mathbf{X}$ their corresponding incidence matrix; $\mathbf{u}$ is the vector of additive genetic random effects; whereas $\mathbf{e}$ is the vector of residuals. It was assumed that random genetic effects follow a normal distribution ${\varvec{u}}\sim N\left(0,{\varvec{G}}{\sigma }_{a}^{2}\right)$ where $\mathbf{G}$ is the genomic relationship matrix (GRM) and ${\sigma }_{a}^{2}$ is the additive genetic variance. The GRM was obtained with VanRaden [28] method as ${\varvec{G}} = \frac{{\user2{ZZ^{\prime}}}}{{2\mathop \sum \nolimits_{j} p_{j} \left( {1 - p_{j} } \right)}},$ where ${p}_{j}$ is the allele frequency of the minor allele (MAF) of marker *j* and $\mathbf{Z}$ the MAF adjusted genotype matrix calculated with the AGHMatrix package [29]. For the estimation of genetic variances with BGLR, default priors were used. The estimate of the narrow-sense heritability was defined as the proportion of variance in the ES explained by additive genetic effects as:

2 $$ {\hat{\mathbf{h}}}_{{\mathbf{a}}}^{{\mathbf{2}}} = {\mathbf{s}}_{{\mathbf{a}}}^{{\mathbf{2}}} /\left( {{\mathbf{s}}_{{\mathbf{a}}}^{{\mathbf{2}}} + {\mathbf{s}}_{{\mathbf{e}}}^{{\mathbf{2}}} } \right) $$

where ${\mathbf{s}}_{\mathbf{a}}^{2}$ and ${\mathbf{s}}_{\mathbf{e}}^{2}$ denote samples of the marginal distribution of the additive genetic and residual variances respectively. The BGLR chain was run for 100,000 iterations, using the first 500 as burn-in and a thinning interval equal to 5. Convergence plots are in Additional file 3 Figure S3A–C. We also converted posterior Gibbs samples, which represent samples of the posterior distribution of the true heritability (${\widehat{\mathbf{h}}}_{\mathbf{a}}^{2})$, into a Monte Carlo Markov Chain (MCMC) object using the as.mcmc() function from the coda package [30] in R. We then used the traceplot() function to generate a trace plot of the MCMC object, which is depicted in Additional file 3 Figure S3D. Finally, for each ${\widehat{\mathbf{h}}}_{\mathbf{a}}^{2}$ we computed the posterior standard deviation ${\varvec{\upsigma}}\left({\widehat{\mathbf{h}}}_{\mathbf{a}}^{2}\right)$ using the coda package in R, as well as the highest posterior density interval 95% (HDP95).

## Results

### Enterosignatures of the pig fecal microbiota

A total of 1755 Amplicon Sequence Variants (ASVs) were identified from the 16S rRNA gene sequence data of the 648 pigs (400 at 60 days and 248 at 190 days). In agreement with previous reports [31, 32], the dominant bacterial phyla were Firmicutes and Bacteroidetes. The optimal rank of ESs was selected by tracking the cosine similarity values reaching a plateau at approximately k = 5. For that value, a minimal fluctuation in the median value was observed, with a total cosine similarity of the original genus abundance around 78% (see Additional file 4 Figure S4A). We estimated the optimal k by computing separate NMF on the whole dataset for k values varying from 2 to 10, saving the corresponding variance explained, cosine similarity, and reconstruction error (see Additional file 4 Figure S4B). The trajectory of k value became steady around the model with five ES explained, which explained 91% variance of the original genus abundances with cosine similarity equal to 95%. Thus, based on this, we propose the existence of at least five ES at 60 days.

**Fig. 1:**
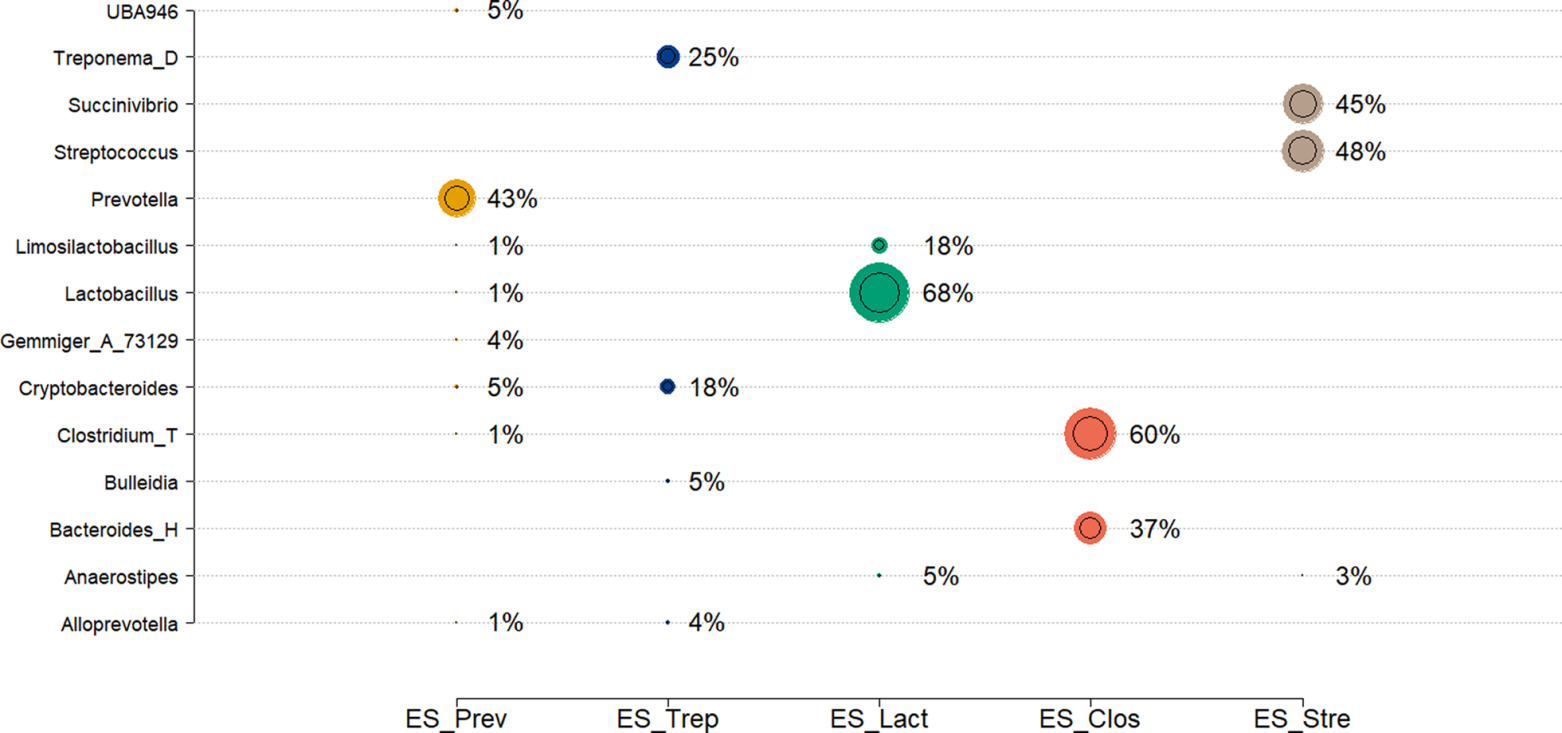
Relative genera composition of pig enterosignatures (ES) dominated by different microbial genera in 60-day-old pigs: ES_Prev, ES_Trep, ES_Lact, ES_Clos, and ES_Stre. Only genera with a contribution higher than 4% in at least one of the ESs are shown

**Fig. 2:**
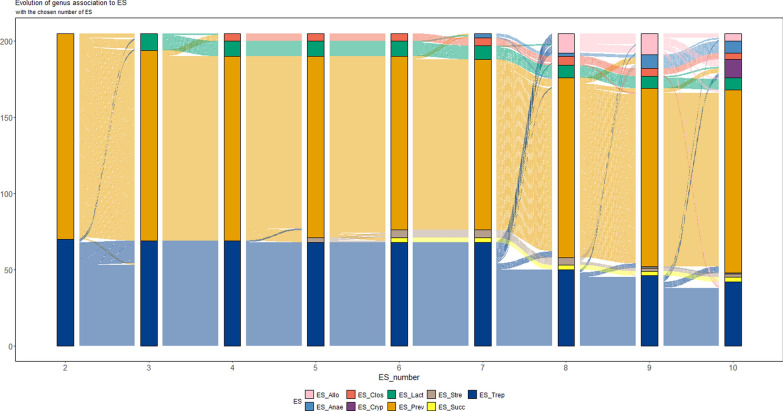
Evolution of genus associations with enterosignatures (ES) for the 400 samples from 60-day-old piglets, depending on the rank of the decomposition

Following the original proposal [14], the driver genus in each ES was identified based on its highest contribution, as is indicated by the normalized **W** matrix. The taxonomic compositions of these five ES were characterized by: ES-Prev (dominated by *Prevotella*), ES-Trep (*Treponema*), ES-Lact (*Lactobaicllus*), ES-Clos (*Clostridium*), and ES-Strep (*Streptococcus*). Figure 1 shows the contents of each ES, displaying only genera with contribution of at least 4% for at least one ES. As summarized in Fig. 1 the combination of bacterial guilds differed dramatically across ESs in terms of genera and composition. For example, *Clostridium* was found at 1% in ES-Prev but at 60% in ES-Clos. Moreover, some ESs such as ES-Clos, were driven by one or two fully dominated genera, while others, such as ES-Trep, were determined by many genera with moderate or weak contributions. Similarly to ES-Clos, ES-Strep was driven by two dominant genera, *Streptococcus* and *Succinivibrio,* with contributions of 48% and 45% respectively. The full composition of the five ESs is in Additional file 5 Figure S5. Particularly, a significant contribution of genera with contributions less than 4% can be seen in ES-Prev and ES-Trep, while in the other three the effect of the driver genera was dominant. In addition, Fig. 2 shows the evolution of the assignment across different k values of genera to each ES. Results show ES-Prev, ES-Trep, and ES-Lact were the most prominent across the different values of k = from 2 to 10, exhibiting the larger prevalence and strongest associations at 60 days of age.

**Fig. 3:**
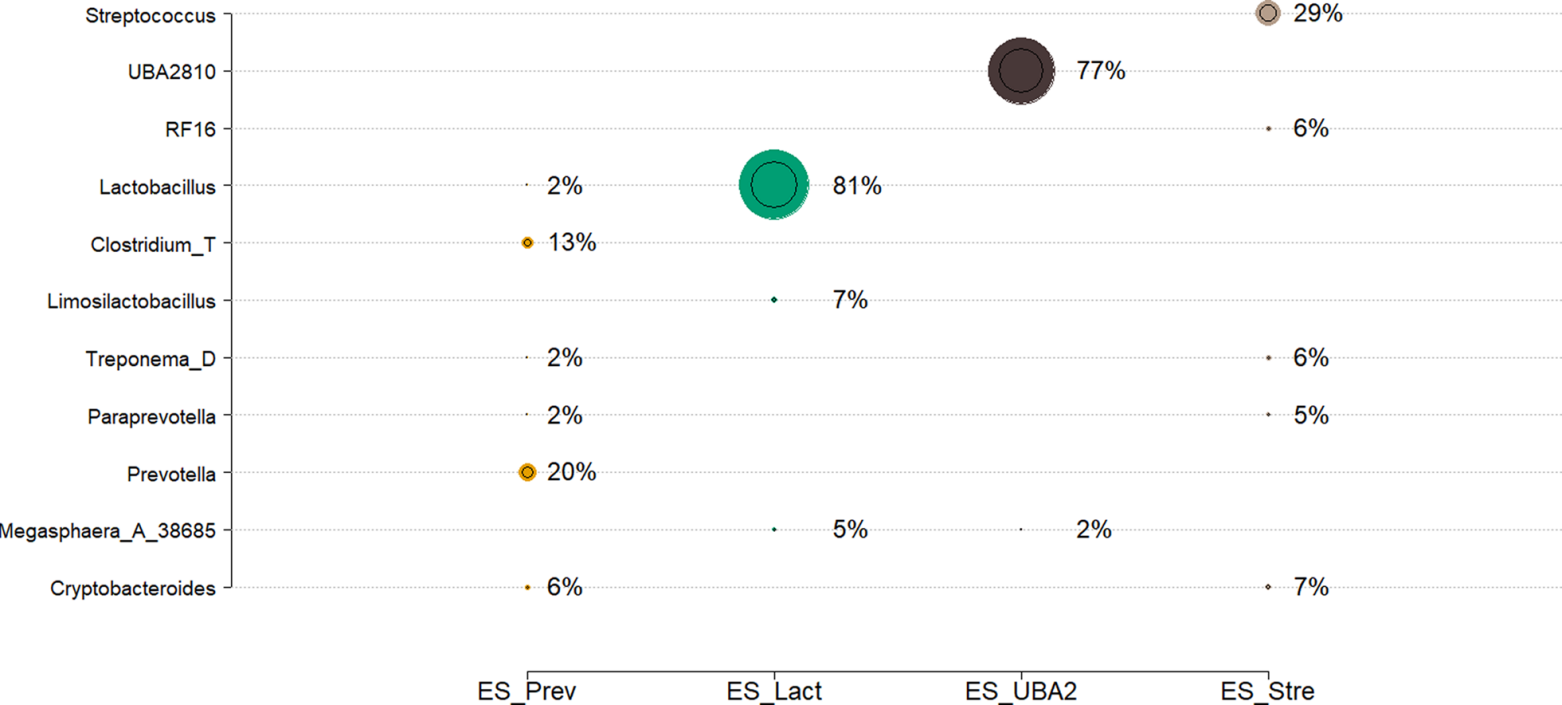
Relative genera composition of enterosignatures (ES) dominated by different microbial genera in 190-day-old pigs: ES_Prev (Prevotella), ES_Lact (Lactobaicllus), ES_UBA2 (UBA2810), and ES_Stre (Streptococcus). Only genera with contribution higher than 4% are shown

Next, we followed the same pipeline to investigate the rank and taxonomic composition of the ES using the dataset of the 248 190-day-old pigs. In this case, the median value of cosine similarity, obtained by bi-cross validation, suggested the optimal NMF solution at k = 4, capturing the 80% of the original matrix (see Additional file 6 Figure S6A). Furthermore, implementation of NMF on the whole data set reported stabilized evaluation scores for four ESs, which explained 87% of the original genus abundances, with cosine similarity equal to 94% (see Additional file 6 Figure S6B). Based on this model, the primary ES driving the variance in the 248 pig microbiota samples were ES-Prev (*Prevotella*), ES-Lact (*Lactobacillus*), ES-UBA2 (*UBA2810*), and ES-Strep (*Streptococcus*). The evolution of genus across the different k values for the 248 samples are in Additional file 7 Figure S7B. The combinations of co-occurring genera and their relative genus-level composition are in Fig. 3, showing that ES-Prev and ES-Strep were characterized by multiple genera with ES-Prev being mainly driven by *Prevotella* and *Clostridium_T*, while ES-Strep was mainly driven by *Streptococcus*. Moreover, ES-Lact and ES-UBA2 were mostly represented by their corresponding dominant genera (*Lactobacillus* and *UBA2810*, respectively). This can be observed in more detail in Additional file 8 Figure S8, where ES-Prev and ES-Strep result in multiple bacteria guilds, whereas ES-Lact and ES-UBA2 represent guilds mainly represented by the dominant taxonomic groups.

### Determinants and consequences of pig faecal ES at 60 days

**Fig. 4:**
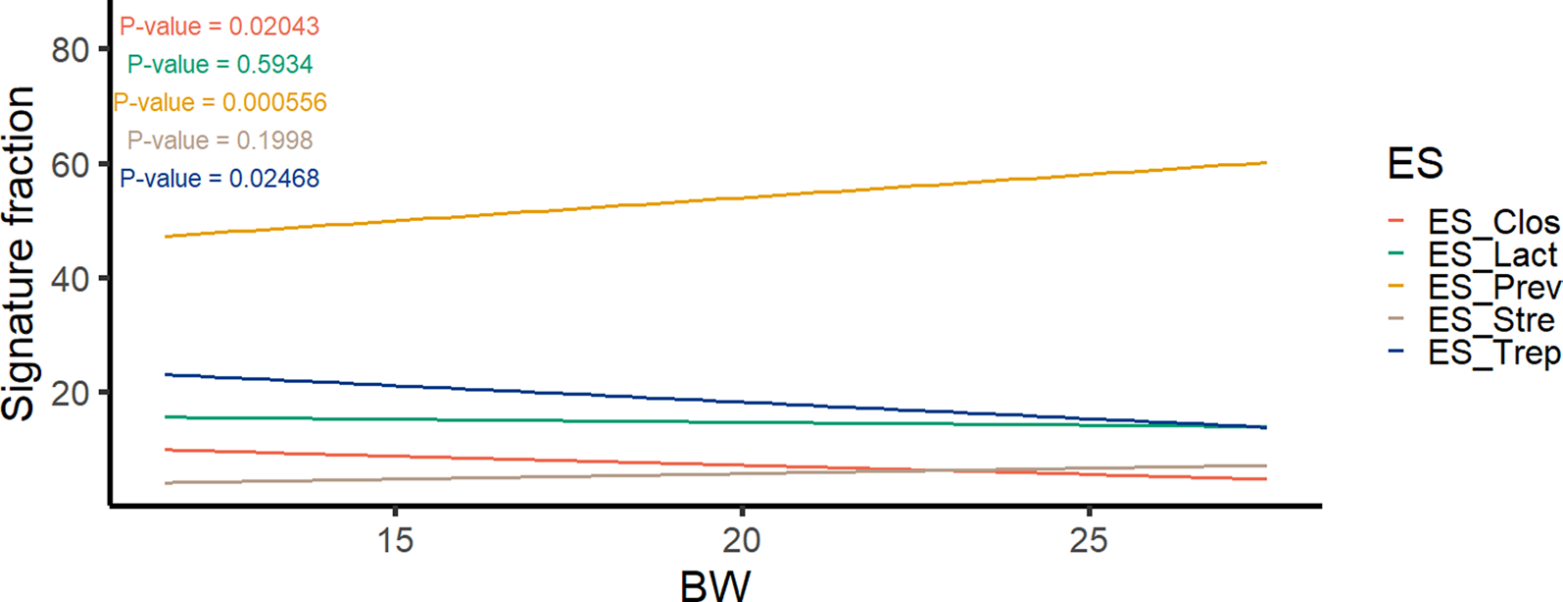
Linear relationship between abundance of enterosignatures (ES) and body weight of 60-day-old pigs. The evolution of ES relative abundances is depicted with the ES faction on the y-axis and BW on the x-axis. P-values are depicted in the legend

Beyond the number of ES and their taxonomic composition, we aimed to understand how ESs are assembled and evolve under the impact of host and environmental factors. We analysed each dataset in relation to different discrete and quantitative traits and factors such as BW, batch, and sex. Specifically, we found significant associations between BW at 60 days and the abundance of ES (Fig. 4). ES-Prev abundance exhibited a strong positive association with BW (P = 0.0006), whereas ES-Trep abundance displayed an opposing pattern (P = 0.025). Moreover, the number and abundance of ESs across the batches of the 400 samples from 60-day-old pigs demonstrated that mostly three ESs were adequate to explain the variance within these samples (see Additional file 9 Figure S9). The primary ES across the different batches appeared to be ES-Prev, followed by ES-Trep and ES-Lact. ES-Strep and ES-Clos were also found to be more prevalent in two out of the six batches. On the other hand, results from assessing sex effects confirmed the same distribution in both males and females (see Additional file 10 Figure S10).

### Determinants and implications of ES during the growing-finishing period

A comparable assessment of sex effects was conducted based on the dataset of 248 190-day-old pigs. Similar to the 60-day-old results, all ESs identified by the NMF model were consistently observed in both females and castrated males (see Additional file 11 Figure S11).

#### Relation with feed efficiency

**Fig. 5:**
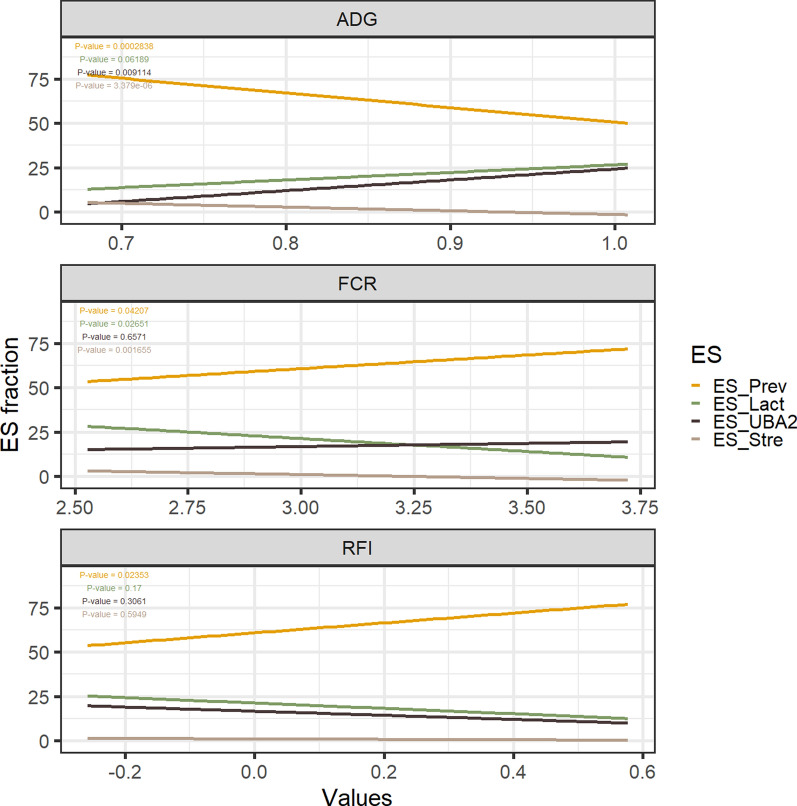
Linear relationship of ESs of 190-day-old pigs with **A** ADG, **B** RFI, and **C** FCR. The relationship of ES relative abundances is depicted with the ES fraction on y-axis and ADG (**A**), FCR (**B**) and RFI (**C**) values in x-axis. P-values are also depicted in the legend at the top-left

The dataset of 134 samples was employed to explore the relationship of ES with ADG and feed efficiency. Abundances of ES-Prev (P = 0.0028) and ES-Strep (P = 3.37E−6) were negatively associated with ADG (Fig. 5). Conversely, positive association trends with ADG were found for ES-Lact (P = 0.061) and ES-UBA (P = 0.009). The negative effect of ES-Prev abundance on feed efficiency was further supported by its positive association with RFI (P = 0.023) and FCR (P = 0.042).

#### Impact of stress on ES and association with cortisol in hair

**Fig. 6:**
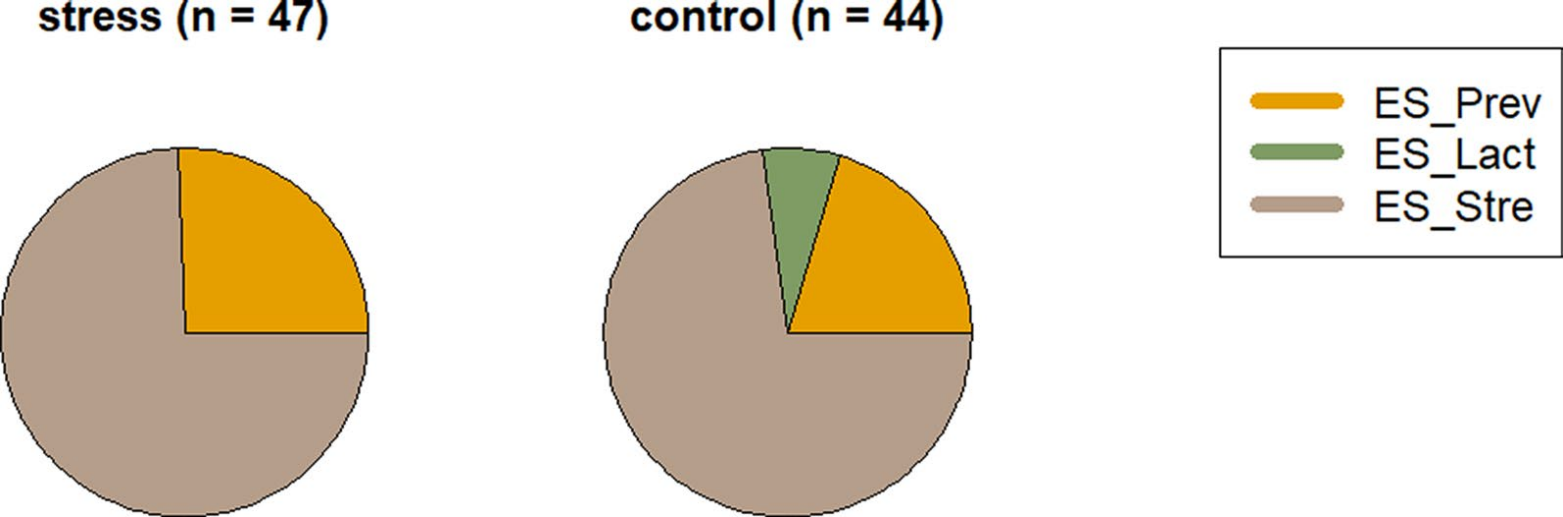
Enterosignatures (ES) composition in stress and control groups of pigs during the growing-finish period

**Fig. 7:**
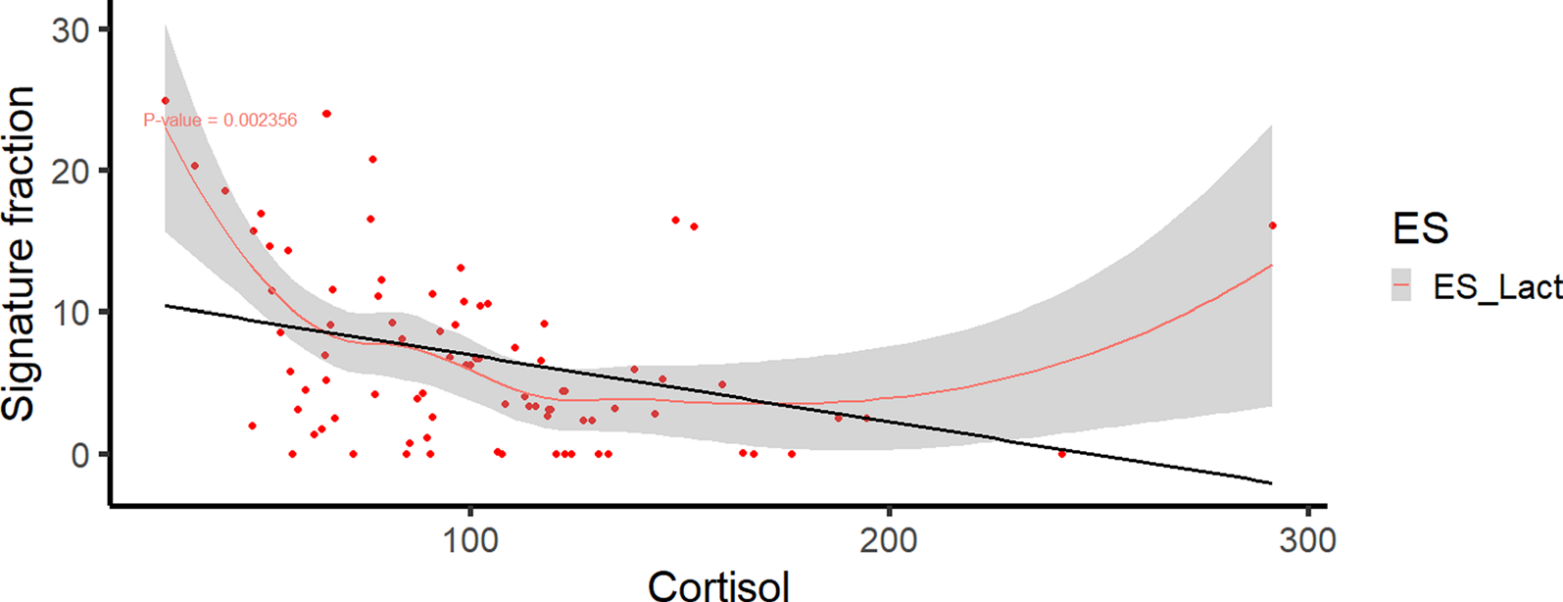
Relationship between ES-Lact abundance and hair cortisol level of 190-day-old pigs. The black line shows the regression line while the red line shows the local regression fit

As stress can influence faecal microbial ecosystems, we investigated the impact of stress during the growing-finishing period on the dominance of ESs (Fig. 6). The largest differences between stress and control groups were characterized by a depletion of ES-Lact and increased abundance of ES-Strep in stressed animals compared to those in the control group. In agreement with this finding, a negative and significant association (P = 0.0023) was found between the abundance of ES-Lact and the levels of the stress-related hormone cortisol in hair (Fig. 7).

### Genetic basis of ES

Table 1 shows the mean, standard deviation (SD), and HDP95 of posterior distributions of heritability for each ES. The highest heritability estimates were observed for ES-Trep [h^2^ = 0.36], followed by ES-Lact [h^2^ = 0.34], ES-Prev [h^2^ = 0.30], ES-Clos [h^2^ = 0.24], and ES-Strep [h^2^ = 0.24]. This suggests that an important fraction of genetic variance in ESs can be explained by the additive genetic value based on SNPs genotypes.

**Table 1:** Summary of posterior mean of heritability (${\widehat{h}}_{a}^{2}$) for each of the enterosignatures (ES) in 60-day-old pigs, along with posterior standard deviation (SD) and 95% highest posterior density interval (HPD95)

	${\widehat{h}}_{a}^{2}$	SD	HPD95 [lower upper]
ES_Prev	0.30	0.0822	[0.14 0.46]
ES_Trep	0.36	0.0760	[0.22 0.51]
ES_Lact	0.34	0.0863	[0.17 0.51]
ES_Clos	0.24	0.0760	[0.10 0.39]
ES_Stre	0.24	0.0645	[0.11 0.37]

## Discussion

Herein, we present the first study investigating the existence of ESs in porcine faecal microbiota. Our results suggest that pig faecal microbiota can be described by combinations of at least six ESs, dominated either by *Prevotella*, *Treponema*, *Lactobacillus*, *Clostridium*, *Streptococcus*, or *UBA2810* genera. Like in humans [14], we observed the dynamic nature of ES in pig microbiota, with shifts occurring in response to the host´s age, i.e. 60- versus 190-day-old pigs. From our observations at the two production stages (transition at 60 days and growing-finishing period at 190 days), we suggest *Prevotella*, *Streptococcus*, and *Lactobacillus* ES as core components. Meanwhile, *Treponema*, *Clostridium*, and *UBA2810* seem to play a time-specific role in the dynamic and assembly of the pig faecal ecosystem. Furthermore, in agreement with previous reports [10], our results confirm strong co-exclusion between *Prevotella* and *Treponema,* which plays a key ecological role in the faecal microbial ecosystem of 60-day-old pigs. We also found abundance of ESs of the 60-day-old pigs to be under partial genetic control of the host, alongside the influence of age and management factors like stress on the assembly of ES for the 190-day-old pigs. Estimates of heritability of ES abundances were found in the range of 0.24 to 0.36, which agreed with other studies in pigs, where low to moderate heritabilities of the abundance of specific genera have been reported [8, 33–36]. Furthermore, in agreement with Larzul et al. [33], abundance of *Treponema*, the main driver of ES-Trep, showed the largest heritability estimate of 0.36.

Regarding the impact of stress (190-day-old pigs), associations were observed between stress-related metrics and ES composition, suggesting potential links. We observed that a prolongated stress challenge can reduce the abundance of ES-Lact, while increasing the prevalence of ES-Strep. Moreover, supporting its positive links with well-being, we observed a negative association between the abundance of ES-Lact and levels of cortisol in hair. Our findings align with previous research, which has shown that *Lactobacillus*, the central genera of ES-Lact, is positively associated with beneficial health outcomes [37, 38]. Numerous studies have also indicated a decrease in relative abundance of *Lactobacillus* under diverse stress challenges, such as heat stress [39–41], weaning, and social stress [37, 42, 43]. Furthermore, research involving various species from the genus *Lactobacillus* and *Limosilactobacillus reuteri* (formerly classified as *Lactobacillus reuteri*) as probiotic supplements has demonstrated a reduction in cortisol levels in plasma and hair within the treated groups when compared to controls [42, 44–46]. This reduction in cortisol levels is attributed to regulation of the hypothalamic–pituitary–adrenal axis and the immune system, as well as the inhibition of inflammatory processes [47–49]. These findings suggest that higher faecal abundance of *Lactobacillus* and *Limosilactobacillus* may contribute to lower cortisol levels in pigs and are consistent with our findings. Therefore, we hypothesize that abundance of ES-Lact could be employed as an indicator of well-being during the growing-finishing period in pigs. Their putative beneficial effect by reducing stress during the porcine growing period deserves further research. Interestingly, greater abundance of *Megasphaera,* another member of ES-Lact, has been associated with lower lesion scores in weaned pigs [50], and less abundant in stressed pigs [51]. ES-Strep was increased in the group of stressed pigs. These results agree well with those of previous studies showing a greater relative abundance of *Streptococcus* and *Treponema* in social stress [51, 52] and in heat stress models [40, 53]. Abundance of *Paraprevotella,* which is also a member of ES-Strep*,* has been proposed as a potential biomarker for depression and other mental disorders in humans [54, 55]. Furthermore, suggesting a potential link with stress, abundance of other co-occurring genera within the ES-Strep, such as *RF16* and *Cryptobacteroides*, have recently been reported as faecal indicators of stress during the growth period of pigs [52].

### Pig enterosignatures are associated with host-performance

We investigated the association of the abundance of ESs with performance traits, focusing on BW at 60 days, and ADG, RFI, and FCR across the growing-finishing period (190-day-old pigs). Consistent with earlier findings by Mach et al. [13] and Ramayo-Caldas. [10], our results confirm the positive association of the abundance of *Prevotella* with BW and with growth rate at 60 days. This association can be attributed to the remarkable capability of *Prevotella* to ferment complex polysaccharides [56] and metabolize dietary fibre, resulting in the production of substantial amounts of short-chain fatty acids (SCFAs), [10]. Interestingly, the *UBA956* genera, which belongs to ES-Prev, also ferments dietary polysaccharides producing SCFAs [57]. This process may contribute to the reduction of intestinal inflammation, thereby ensuring improved intestinal absorption capacity and increasing feed efficiency [58].

Our results also confirmed a negative association of the abundance of *Streptococcus* [59, 60] and the positive of the abundance of ES-Lact [61, 62] with growth and feed efficiency during the growing-finishing period (190-day-old pigs). Intriguingly, although *Prevotella* is known to be beneficial after weaning, our results showed that, together with ES-Strep, the abundance of ES-Prev was negatively associated with ADG during the growing-finish period. Additionally, ES-Prev abundance exhibited an unfavourable association with feed efficiency, displaying a positive association with RFI and FCR. In agreement with our findings, an increased abundance of *Prevotella copri* in pigs with lower efficiency has been reported by Jiang et al. [63]. Moreover, *Prevotella copri* has been shown to increase fat deposition in Duroc pigs [64]. In growing pigs, the growth of lean tissue demands less energy than fat deposition, leading to a more efficient transformation of feed into muscle on a weight basis. Consequently, we hypothesise that the association of *Prevotella* abundance with fat deposition during the growing period is partially explained by the observed adverse association between ES-Prev abundance and feed efficiency.

### Can the ES concept be integrated into breeding programs and precision farming?

Modulating the gut microbiome represents a promising strategy for enhancing animal welfare and productivity, thus contributing to livestock sustainability. This can be achieved through a combination of breeding and nutritional strategies. As demonstrated by Frioux et al. [14], and supported by our findings, microbial ecosystems can be accurately described by combinations of ESs, which, in our study, explained up to 91% of the variance in the original genera abundance. Therefore, ES can reduce the complexity of microbial ecosystems, featuring microbial assemblies that are relevant for practical applications. We also showed the heritable nature of pig faecal ES composition and its association with host performance. Thus, pig ESs meet two crucial criteria for integration into breeding programs, potentially improving the prediction of breeding values and host performance, while also supporting microbiome-based precision farming. We hypothesize that ES be a valuable tool for detecting microbial perturbations to monitor productivity, animal welfare, and health. However, our findings highlight the importance of developing microbial consortiums tailored to the nutritional and health needs of pigs at each production stage. The observed association patterns for ES-Prev are a good example that what might benefit during transition may not be favourable during the pig growing-finishing period. Young piglets likely require microbial support for immune development and adaptation to cereal-based diets post-weaning, while during the growing-finishing period, older pigs might benefit from a different combination of strains that enhance nutrient absorption, protein breakdown, and gut health. Therefore, we anticipate that without evidence-based recommendations regarding the safety and efficacy of pre/pro/postbiotic formulations for specific stages, microbiome-based strategies will have limited success in improving health and performance in livestock production.

We would like to recognize here some limitations of our study, particularly that our model is based on a dataset of a Duroc pig population at 60 days and at the end of the growing-finishing period (190 days). As a result, it is reasonable to assume that ES for other porcine breeds, developmental stages, or management conditions are not captured by our results. Consequently, we anticipate that combinations of additional microbial guilds might be of relevance in the assembly of pig faecal microbial ecosystems in other situations. Despite these limitations, our findings provide novel insights into the pig gut microbiota, including the dynamic changes in ES assemblies with host age, their connection to stress, and part control by host genetics. Additionally, we demonstrated associations of the abundance of ES with host physiology and productive performance, suggesting the usefulness of the ES concept in animal breeding.

## Conclusions

The pig faecal microbiota of our Duroc population was characterized by a combination of at least six ESs, dominated by either P*revotella*, *Treponema*, *Lactobacillus*, *Clostridium*, *Streptococcus*, or *UBA2810*. ES assemblies changed dynamically with the age of the host, were connected to stress, and in part shaped by host genetics. Relevant associations between the abundance of ES communities and host productive performance were identified. Overall, our findings offer novel insights into the pig gut microbiota assembly, suggesting the usefulness of the ES concept in breeding and precision farming, but also underscoring the relevance of customizing microbial consortia based on the nutritional and health requirements at each stage of the porcine production cycle.

## Supplementary Information

**Additional file 1: Figure S1.** Experimental design of samples employed in the study.

**Additional file 2: Figure S2.** Approach of enterosignatures (ES) applying NMF to determine bacteria guilds driving variance in pig gut microbiota.

**Additional file 3: Figure S3.** Plot shows (A) residual variances, (B) genetic variances and (C) heritability estimates across iterations to show convergence. (D) precents the trace plot of h2 after converting the BGLR Gibbs samples of h2 estimates into a MCMC object using the coda package. Results correspond to “ES-Trep” under model (1).

**Additional file 4: Figure S4.** (A) Explained variance of the original genus abundances for the 400 samples applying 3x3 bi-cross validation for a k range from 2 to 30. (B) Figure displays the scores for three evaluation metrics such as explained variance, cosine similarity and reconstruction error for k varies from 2 to 10.

**Additional file 5: Figure S5.** Pie plots show the genus-level composition of the five ESs for the 400 samples (60-days old) classified as “others” what is below 4%.

**Additional file 6: Figure S6.** (A) Explained variance of the original genus abundances for the 248 samples (190-days old) applying 3x3 bi-cross validation for a k range from 2 to 30. (B) Figure displays the scores for three evaluation metrics such as explained variance, cosine similarity and reconstruction error for k varies from 2 to 10.

**Additional file 7: Figure S7.** Evolution of genus association to pig enterosignatures (ES) for the 248 samples (190-days old), depending on the rank of the decomposition.

**Additional file 8: Figure S8.** Pie plots show the genus-level composition of the four ESs for the 248 samples classified as “others” what is below 4%.

**Additional file 9: Figure S9.** Pie plots show the six batch groups and their composition in terms of number of ESs and ES name for the 400 samples at 60-days. Grey scale pie plots indicate the frequency of the minimal number of ES number needed to explain most of the variance in the samples. Multi-coloured plots show the frequency of each ES as primary ES in each group.

**Additional file 10: Figure S10.** Pie plots show the two groups resulted by sex category and their composition in terms of number of ESs and their name at 60-days. Grey scale pie plots indicate the frequency of the minimal number of ES number needed to explain most of the variance in the samples. Multicoloured plots show the frequency of each ES as primary ES in each group.

**Additional file 11: Figure S11.** Pie plots show the two groups resulted by sex category and their composition in terms of number of ESs and their name for the 248 pigs sampled at 190-days. Grey scale pie plots indicate the frequency of the minimal number of ES number needed to explain most of the variance in the samples. Multi-coloured plots show the frequency of each ES as primary ES in each group.

## Data Availability

The raw sequencing data employed in this article has been submitted to the NCBI’s Sequence Read Archive (https://www.ncbi.nlm.nih.gov/sra); and BioProject under accession number: PRJNA608629.
