# Supplementary figures and images for: Co-occurring microbial guilds in pig fecal microbiota: key drivers and effects on host performance
Source: Genet Sel Evol. 2025 Jun 4;57:27. doi: 10.1186/s12711-025-00979-x (PMC12139276; doi:10.1186/s12711-025-00979-x)

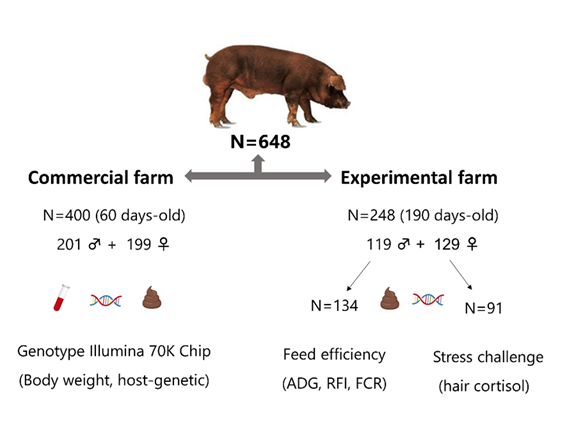

Supplement: Supplementary file 1 — Additional file 1: Figure S1. Experimental design of samples employed in the study. [file 12711_2025_979_MOESM1_ESM.png]

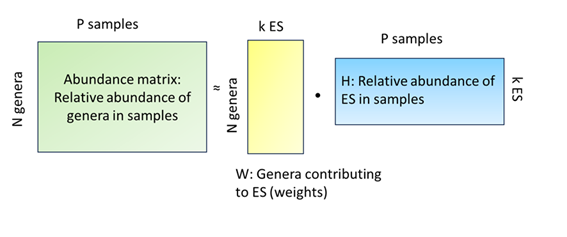

Supplement: Supplementary file 2 — Additional file 2: Figure S2. Approach of enterosignatures (ES) applying NMF to determine bacteria guilds driving variance in pig gut microbiota. [file 12711_2025_979_MOESM2_ESM.png]

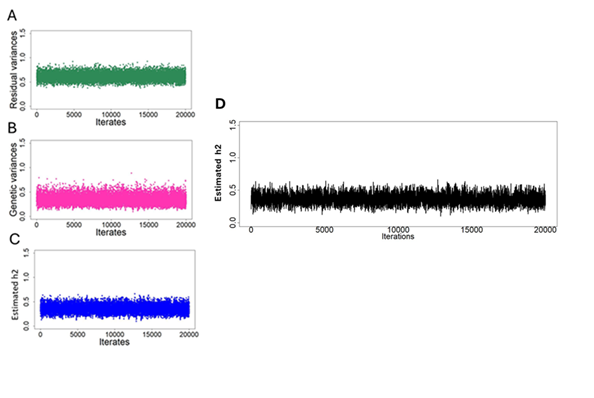

Supplement: Supplementary file 3 — Additional file 3: Figure S3. Plot shows (A) residual variances, (B) genetic variances and (C) heritability estimates across iterations to show convergence. (D) precents the trace plot of h2 after converting the BGLR Gibbs samples of h2 estimates into a MCMC object using the coda package. Results correspond to “ES-Trep” under model (1). [file 12711_2025_979_MOESM3_ESM.png]

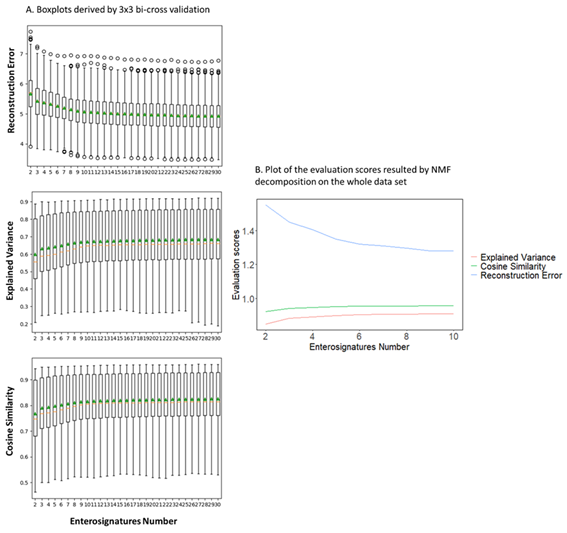

Supplement: Supplementary file 4 — Additional file 4: Figure S4. (A) Explained variance of the original genus abundances for the 400 samples applying 3x3 bi-cross validation for a k range from 2 to 30. (B) Figure displays the scores for three evaluation metrics such as explained variance, cosine similarity and reconstruction error for k varies from 2 to 10. [file 12711_2025_979_MOESM4_ESM.png]

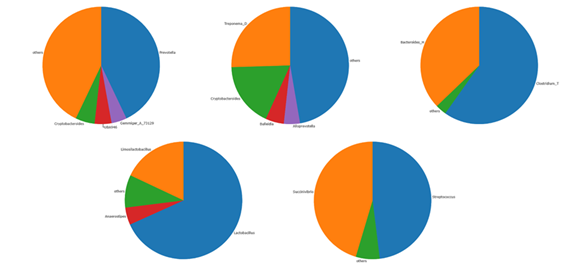

Supplement: Supplementary file 5 — Additional file 5: Figure S5. Pie plots show the genus-level composition of the five ESs for the 400 samples (60-days old) classified as “others” what is below 4%. [file 12711_2025_979_MOESM5_ESM.png]

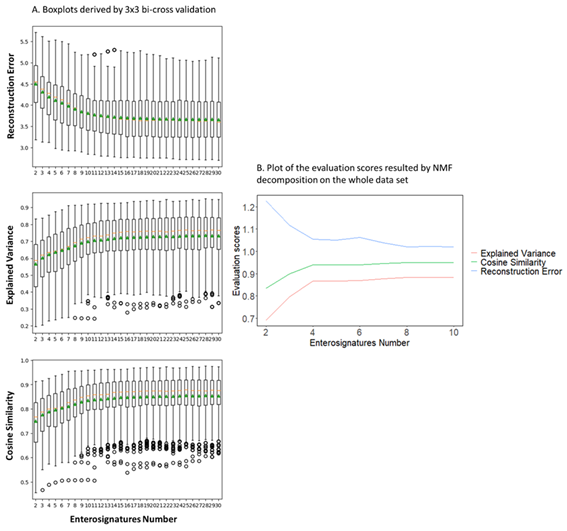

Supplement: Supplementary file 6 — Additional file 6: Figure S6. (A) Explained variance of the original genus abundances for the 248 samples (190-days old) applying 3x3 bi-cross validation for a k range from 2 to 30. (B) Figure displays the scores for three evaluation metrics such as explained variance, cosine similarity and reconstruction error for k varies from 2 to 10. [file 12711_2025_979_MOESM6_ESM.png]

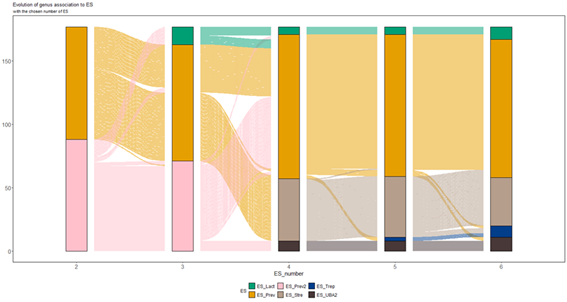

Supplement: Supplementary file 7 — Additional file 7: Figure S7. Evolution of genus association to pig enterosignatures (ES) for the 248 samples (190-days old), depending on the rank of the decomposition. [file 12711_2025_979_MOESM7_ESM.png]

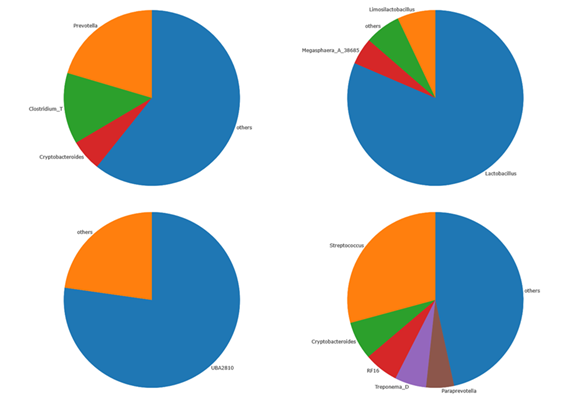

Supplement: Supplementary file 8 — Additional file 8: Figure S8. Pie plots show the genus-level composition of the four ESs for the 248 samples classified as “others” what is below 4%. [file 12711_2025_979_MOESM8_ESM.png]

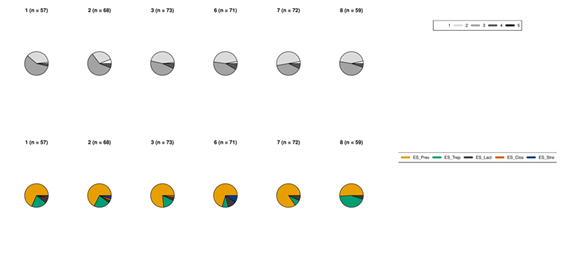

Supplement: Supplementary file 9 — Additional file 9: Figure S9. Pie plots show the six batch groups and their composition in terms of number of ESs and ES name for the 400 samples at 60-days. Grey scale pie plots indicate the frequency of the minimal number of ES number needed to explain most of the variance in the samples. Multi-coloured plots show the frequency of each ES as primary ES in each group. [file 12711_2025_979_MOESM9_ESM.png]

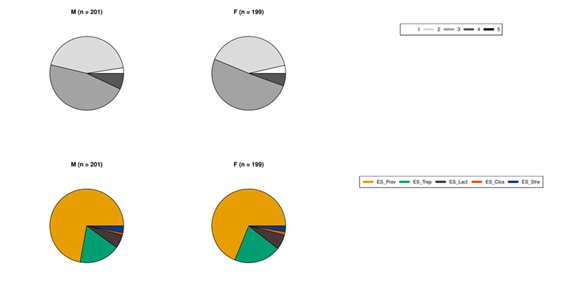

Supplement: Supplementary file 10 — Additional file 10: Figure S10. Pie plots show the two groups resulted by sex category and their composition in terms of number of ESs and their name at 60-days. Grey scale pie plots indicate the frequency of the minimal number of ES number needed to explain most of the variance in the samples. Multicoloured plots show the frequency of each ES as primary ES in each group. [file 12711_2025_979_MOESM10_ESM.png]

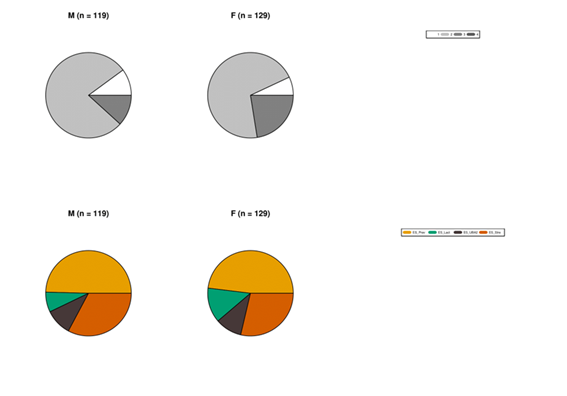

Supplement: Supplementary file 11 — Additional file 11: Figure S11. Pie plots show the two groups resulted by sex category and their composition in terms of number of ESs and their name for the 248 pigs sampled at 190-days. Grey scale pie plots indicate the frequency of the minimal number of ES number needed to explain most of the variance in the samples. Multi-coloured plots show the frequency of each ES as primary ES in each group. [file 12711_2025_979_MOESM11_ESM.png]
